# Ameliorative Effect of Curcumin-Encapsulated Hyaluronic Acid–PLA Nanoparticles on Thioacetamide-Induced Murine Hepatic Fibrosis

**DOI:** 10.3390/ijerph14010011

**Published:** 2016-12-24

**Authors:** Yu-Nong Chen, Shih-Lan Hsu, Ming-Yuan Liao, Yi-Ting Liu, Chien-Hung Lai, Ji-Feng Chen, Mai-Huong Thi Nguyen, Yung-Hsiang Su, Shang-Ting Chen, Li-Chen Wu

**Affiliations:** 1 Department Applied Chemistry, College of Science and Technology, National Chi Nan University, Nantou, Puli 545, Taiwan; s99324534@ncnu.edu.tw (Y.-N.C.); s98327510@ncnu.edu.tw (Y.-T.L.); a723713a@hotmail.com (C.-H.L.); rosylala@gmail.com (J.-F.C.); nguyenthimaihuong1989@gmail.com (M.-H.T.N.); willy51118@gmail.com (Y.-H.S.); s104324522@mail1.ncnu.edu.tw (S.-T.C.); 2 Department Medical Education & Research, Taichung Veterans General Hospital, Taichung 40705, Taiwan; h2326@vghtc.gov.tw; 3 Department Chemistry, College of Sciences, National Chung Hsing University, Taichung 402, Taiwan; mliao@dragon.nchu.edu.tw

**Keywords:** hyaluronic acid, curcumin, nanoparticle, hepatic stellate cells, liver fibrosis

## Abstract

In this study, we developed curcumin-encapsulated hyaluronic acid–polylactide nanoparticles (CEHPNPs) to be used for liver fibrosis amelioration. CD44, the hyaluronic acid (HA) receptor, is upregulated on the surface of cancer cells and on activated hepatic stellate cells (aHSCs) rather than normal cells. CEHPNPs could bind to CD44 and be internalized effectively through endocytosis to release curcumin, a poor water-soluble liver protective agent. Thus, CEHPNPs were potentially not only improving drug efficiency, but also targeting aHSCs. HA and polylactide (PLA) were crosslinked by adipic acid dihydrazide (ADH). The synthesis of HA–PLA was monitored by Fourier-transform infrared (FTIR) and Nuclear Magnetic Resonance (NMR). The average particle size was approximately 60–70 nm as determined by dynamic light scattering (DLS) and scanning electron microscope (SEM). Zeta potential was around −30 mV, which suggested a good stability of the particles. This drug delivery system induced significant aHSC cell death without affecting quiescent HSCs, hepatic epithelial, and parenchymal cells. This system reduced drug dosage without sacrificing therapeutic efficacy. The cytotoxicity IC_50_ (inhibitory concentration at 50%) value of CEHPNPs was approximately 1/30 to that of the free drug treated group in vitro. Additionally, the therapeutic effects of CEHPNPs were as effective as the group treated with the same curcumin dose intensity in vivo. CEHPNPs significantly reduced serum aspartate transaminase/alanine transaminase (ALT/AST) significantly, and attenuated tissue collagen production and cell proliferation as revealed by liver biopsy. Conclusively, the advantages of superior biosafety and satisfactory therapeutic effect mean that CEHPNPs hold great potential for treating hepatic fibrosis.

## 1. Introduction

Hepatic fibrosis that is caused by chronic damage, due to alcohol abuse, nonalcoholic steatohepatitis (NASH), and virus infection (e.g., hepatitis C virus (HCV)) accumulate excessive extracellular matrix proteins through the promoted, wound-healing process [1]. Collagen produced by activated hepatic stellate cells (aHSCs) disturbs normal hepatic function and architecture. Fibrosis often leads to portal hypertension or cirrhosis because there are no severe symptoms. Quiescent hepatic stellate cells (qHSCs) store vitamin A and mediate retinoid-associated homeostasis, and they also regulate portal venous pressure of hepatic blood flow. Once activated, HSCs start the progress of hepatic fibrosis [2] and undergo phenotypic and morphologic changes (myofibroblast-like) that include enhanced proliferation and motility, and production of cytokines, type I collagen, matrix metalloproteinase (MMP), and α-smooth muscle actin [3]. Cytokines such as tumor necrosis factor-alpha (TNF-α) and interleukins secreted by Kupffer cells, hepatocytes, and leukocytes elicit the activation of HSCs further. Autocrine and paracrine signaling loops perpetuate proliferation, chemotaxis, and fibrogenesis [4].

HSCs are described as liver-specific pericytes for angiogenesis and vascular remodeling during fibrosis [5]. These cells drive sinusoidal vascular remodeling through paracrinal cross-talk with liver sinusoidal endothelial cells (LSECs) by releasing angiogenic cytokines, and recruiting LSECs to the vascular wall [6]. Platelet-derived growth factor (PDGF) that is secreted by LSECs plays a key role in vascular remodeling. This fibrogenic cytokine stimulates the production of proangiogenic molecules of aHSCs and promotes aHSC motogenic activity to mobilize around vessel walls [7]. Additionally, the increased expression of CD44, a hyaluronic acid cell surface receptor, facilitates the migration of aHSCs [8].

Hyaluronic acid (HA), a nonsulfated glycosaminoglycan (GAG) that is distributed in extracellular matrix, consists of repeated disaccharides of α-1,4-d-glucuronic acid and β-1,3-*N*-acetyl-d-glucosamine [9,10,11]. The molecular weight of HA ranges widely from 10^3^ to 10^7^ Da [11]. HA interacts with CD44, the main cell surface HA receptor, to undergo various cellular activities that include cell proliferation, differentiation, migration, survival, angiogenesis, molecule release (cytokine/chemokine/growth factor), and protease docking at the cell membrane [12]. Other HA-binding receptors, such as the hyaluronan receptor for endocytosis (HARE), the receptor for HA-mediated motility (RHAMM or CD168), and the intracellular adhesion molecule-1 (ICAM-1, or CD54) are reported, and are suggested to initiate distinct signaling cascades [13].

HA-based therapeutic strategies have been applied in cancer treatments [14]. The physicochemical and biological properties of HA make this extracellular matrix material preferable for drug delivery applications and it is applied in cancers [14,15]. These bioinert, biodegradable, and biocompatible polysaccharides conjugate with drugs or constitute polymeric nanocomplexes/nanoparticles to deliver hydrophobic anticancer drugs or small interfering RNA (siRNA) to target cancer cells, which overexpress CD44 and isoforms [15]. Interestingly, high-molecular-weight HA (HMW-HA) is inert and functions as a structural polysaccharide, whereas low-molecular-weight (LMW-HA) or HA oligomers stimulate inflammatory reaction, proangiogenesis, and cell proliferation and migration [16]. Accordingly, the use of bioinert HMW-HA in nanoformulation is applied widely.

Recently, the applications of natural product-based nanomedicine to improve the bioavailability of poor water-soluble compounds and to enhance therapeutic efficacy have been suggested [17]. These natural product-encapsulated nanodrugs gain success in vitro and in vivo by reducing adverse side effects by cutting the dosage, but also by increasing the efficacy of natural products through specific targeting [18]. However, the potential nanotoxicity of natural product-based targeting systems remains a challenge, especially for the treatment for chronic diseases. Therefore, the biocompatibility of the drug carrier, the side effects of natural products, and drug dosage should be considered when developing natural product-based nanoformulations. HA holds excellent biocompatibility and can be a candidate as a drug carrier.

Several natural products have been proven to be potent agents for liver protection [19]. Curcumin, an active diarylheptanoid of the turmeric rhizome *Curcuma longa*, possesses anti-inflammatory effects that retard hepatic fibrosis and cirrhosis in carbon tetrachloride (CCl4)-caused liver fibrosis [20]. This herbal antioxidant alleviates fibrosis through the effects of reduced activation, retarded mobility, and angiogenetic activity on HSCs [21]. Curcumin suppresses HSC activation through disruption of transforming growth factor-β (TGF-β) signaling [22] and activation of peroxisome proliferator-activated receptor-c (PPAR-c) [23]. Activated PPAR-c signaling not only interrupts focal adhesion kinase (FAK)/RhoA, extracellular signal-regulated kinases (ERKs), and mechanistic target of rapamycin (mTOR) signaling cascades, but also downregulates the expression of PDGF-β receptor (PDGF-βR), thereby limiting HSC-based vascularization [21].

Even though curcumin has drawn significant attention because of its antifibrotic activity and its safety [24], the issues of insolubility in water, non-absorption/bioavailability, and rapid metabolism and excretion make it problematic in Phase I clinical trials [25]. Approaches that increase curcumin bioavailability have been suggested, and nanomedicine appears to be a feasible way to increase the efficacy of curcumin [26]. Herein, we proposed a nanoparticle delivery system using HA–polylactide (PLA) that is encapsulated with curcumin for the amelioration of rat hepatic fibrosis. As mentioned already, aHSC is critical to the progression of fibrosis and expresses a high level of CD44. It would be rational to target aHSCs by using an HA-based therapeutic strategy. Our results should provide insights into an HA-based curcumin nanomedicine therapeutic strategy on fibrosis amelioration.

## 2. Materials and Methods

### 2.1. Materials

Adipic acid dihydrazide (ADH), coumarin-6, dimethyl sulfoxide (DMSO), *N*-(3-dimethylaminopropyl)-*N’*-ethylcarbodiimide hydrochloride (EDC), and thioacetamide (TAA) were obtained from Sigma-Aldrich Co. (St. Louis, MO, USA). DMSO (extra dry) was purchased from Acros Co. (Geel, Belgium). *N*-hydroxysuccinimide (NHS) and *N*,*N’*-dicyclohexylcarbodiimide (DCC) were obtained from Fluka Ltd. (Buchs, Switzerland). Poly(d,l)-lactide (M_w_ 16,000–35,000 Da) was purchased from Green Square (Taichung, Taiwan). Hyaluronic acid was provided by Dr. Shih-lan Hsu. All other reagents were of the highest grade commercially available.

### 2.2. Acid Hydrolysis of HA

HA (2 g in 183 mL deionized distilled water (ddH_2_O)) was hydrolyzed by 1.5 M HCl (pH 3.0 for 12 h). The pH of hydrolyzed HA was adjusted to 7.0 with 12 M NaOH at room temperature. The HA solution was dialyzed against ddH_2_O with SnakeSkin Pleated Dialysis Tubing (M_w_ cutoff (MWCO) 3500 Da, Thermo Fisher Scientific, Waltham, MA, USA) for 24 h with consistent changing of dialysis water. The molecular weight was analyzed by gel permeation chromatography (GPC). The low-molecular-weight HA (M_w_ 10^3^ Da) was freeze-dried into powder and kept in desiccators until use.

### 2.3. Gel Permeation Chromatography (GPC)

The GPC system (Waters, Milford, MA, USA) consisted of an high performance liquid chromatography (HPLC) pump, an injection valve with a 200 μL sample loop, three hydroxylated polymethacrylate-based gel columns, and a refractive index detector with a temperature module set at 35 °C. GPC tandem columns (pore sizes 0.2 and 0.025 μm, 7.8 × 300 mm) (Waters, Milford, MA, USA) were used for separation. The flow rate was 0.7 mL/min. The mobile phases were ammonium phosphate (0.02 M, pH 4.5), potassium phosphate (0.02 M, pH 6.9), and Tris-HCl buffer (0.02 M, pH 9.5). Samples were freshly prepared before injection. A calibration curve was constructed by Empower 2 chromatography data software (Waters, Milford, MA, USA) using dextran as standards.

### 2.4. Synthesis of HA–ADH

**Figure 1:**
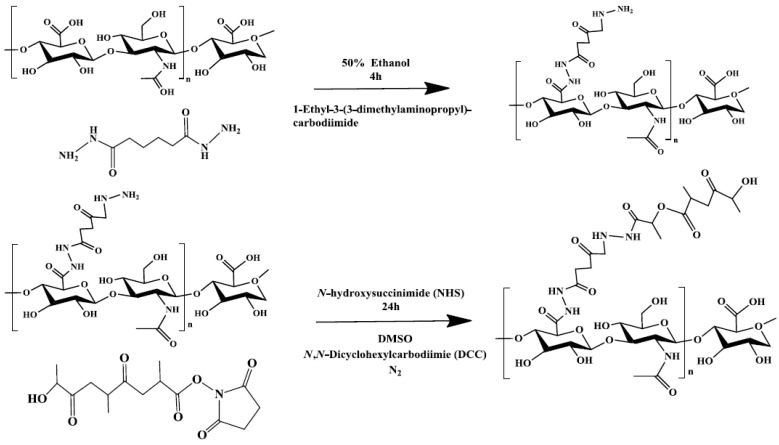
The procedures for synthesizing hyaluronic acid–adipic acid dihydrazide–polylactide (HA–ADH–PLA). DMSO: dimethyl sulfoxide.

The synthesis of HA–ADH (Figure 1) followed the protocol of Park et al. [27]. Briefly, ADH (4~5 g) was dissolved in ddH_2_O (40 mL). The hydrolyzed hyaluronic acid sample (M_W_: 103 Da) was dissolved in ddH_2_O in another beaker to obtain the final concentration of 5 mg/mL. HA was dropped slowly into ADH solution and stirred for 30 min, followed by adjusting pH to 4.8 to terminate the reaction. An equal amount of ethanol was then added to the HA–ADH solution. After stirring the ethanol/HA–ADH mixture for 1 h, EDC (0.4 g) was added to the resultant solution at pH 4.8 for another 2 h. The mixture was neutralized by the addition of NaOH (12 M) to stop the reaction. The solution was dialyzed for 3 days and then freeze-dried into powder.

### 2.5. Synthesis of HA–ADH–PLA

The synthesis of HA–ADH–PLA (Figure 1) followed the protocol of Park et al. [27]. Briefly, HA–ADH (230 mg) was added to DMSO (50 mL) and stirred for 30 min. PLA (1 g), DCC (99 mg), and NHS (55 mg) were dissolved in another 50 mL of DMSO. These two solutions were mixed and stirred for 24 h. The resultant solution was dialyzed by SnakeSkin Pleated Dialysis Tubing (50,000 Da MWCO) for 3 days and then freeze-dried. The sample was dissolved in acetone and then subjected to vacuum concentration to remove acetone to obtain HA–ADH–PLA powder.

### 2.6. Preparation of HA–ADH–PLA Nanoparticles

The drug (curcumin or coumarin-6, 0.2 mg) was dissolved completely in 10 mL of acetone in ultrasonic. HA–ADH–PLA (15 mg) that was dissolved in acetone was then added to the drug solution. The mixture was dropped (25 mL/h) into 40 mL ddH_2_O, ultrasonicated for 1 min, and then stirred overnight at ambient temperature in the dark. The solution was under vacuum to remove acetone. Particles were resuspended by ddH_2_O (20 mL), and filtered through a 0.2 μm pore size syringe filter. Particles were kept in 4 °C until use.

### 2.7. Measurement of Particle Size and Zeta Potential

The sample was diluted in ddH_2_O and filtered through a 0.22 μm pore size syringe filter. The particle size was measured by a N4 Plus PCS Submicron Particle Size Analyzer (Beckman Coulter, Palo Alta, CA, USA). The zeta potential of nanoparticles was analyzed by Zetasizer (Malvern Nano Series, Worcetershire, UK). Particle morphology was analyzed by afield-emission scanning electron microscope (JEOL JSM-6700F, Tokyo, Japan).

### 2.8. Analysis of Drug Encapsulation Analysis

After the preparation of curcumin-encapsulated HA–PLA nanoparticles (CEHPNPs), the solution was centrifuged, and the supernatants were collected for the detection of unincorporated drug by a spectrophotometer (Beckman Coulter) at 450 nm, and the pellets were freeze-dried, followed by dissolving CEHPNPs in acetone to extract curcumin for the calculation of loading efficiency. The entrapment efficiency (E.E.) and the loading efficiency (L.E.) of the nanoparticles were calculated as follows:

Entrapment efficiency (%) = (Weight of drug fed − Free drug)/(Weight of drug fed) × 100

Loading efficiency (%) = (Weight of drug in nanoparticles)/(Weight of drug and polymer) × 100

### 2.9. Analysis of Releasing Profile Analysis

The drug-releasing profile of HA–PLA nanoparticles was measured at pH 5.0 (acetate buffer) and 7.4 (phosphate buffer). CEHPNPs were added to a dialysis bag (3500 Da MWCO). The bag was sealed and placed in a beaker that contained containing 100 mL of acetate buffer or phosphate buffer with consistent stirring. Samples were taken every 5 h and then replaced with the same volume of buffer. Curcumin was measured by a spectrophotometer (Beckman Coulter) at 450 nm.

### 2.10. Cell Culture, Cell Viability, and Cytotoxicity

HSCs were isolated from rat liver tissues and cultured in Dulbecco’s Modified Eagle’s medium (DMEM) (Gibco, Thermo Fisher Scientific, Waltham, MA, USA) that contained 10% fetal bovine serum (FBS) and 1% antibiotics, and it was then grown in a 5% CO_2_-humidified atmosphere at 37 °C. HSCs that were subcultured three times were used in experiments. For cell viability, HSCs (5 × 10^4^ cells/well) were seeded in 96-well plates (NUNC, Roskide, Denmark) and then cultured in DMEM or endothelial cell medium with 10% FBS for 24 h. Cells were treated with different reagents for 4 h and then incubated for 12 h at 37 °C. The MTT (3-(4,5-dimethylthiazol-2-yl)-2,5-diphenyltetrazolium bromide) cell proliferation assay kit (Sigma, St. Louis, MO, USA) was used to determine cell viability [28]. Absorbance was read at 570 nm.

### 2.11. Expression of CD44

The expression of CD44 in MDA-MB-231, A549, aHSCs, qHSCs, ZR-75-1, and clone 9 cells was analyzed by a FACSCalibur system (BD Biosciences, Franklin Lakes, NJ, USA) using CellQuest software (Franklin Lakes, NJ, USA). Data were representative of at least three independent experiments.

### 2.12. Experimental Animals

All procedures followed the Guide for the Care and Use of Laboratory Animals of Taiwan Animal Protect Act and the Committee on the Ethics of Animal Experiments of National Chi Nan University (approval number: 1020528-1). Male balb/c mice (25 g body weight) were purchased from BioLASCO (Yilan, Taiwan). A mixture of TAA (50 mg/kg body weight, intraperitoneally (i.p.), three times/week) was used to induce rat liver fibrosis for 9 weeks. Twenty mice were randomly separated into five groups (4 mice/group). Group 1 was the control in which mice were not administrated TAA or curcumin or nanoparticles. Except group 1, mice in other groups were treated with TAA. Group 2 was the TAA control, in which mice received no drug treatment. Groups 3 and 4 were injected (i.p.) with free curcumin at 20 and 150 mg/kg·wt, respectively. Group 5 was treated with CEHPNPs at a dosage of curcumin at 20 mg/kg·wt (i.p.). Weights of mice were recorded. Mice were sacrificed after 8 weeks of drug treatment by heart bleeding under anesthesia. Blood and livers were collected for analysis. A small amount of livers was taken for histopathological and immunohistochemical examination. Remaining liver tissues were frozen in liquid nitrogen.

### 2.13. Liver Histopathological Analysis and Measurement of Serum Aspartate Transaminase/Alanine Transaminase (ALT/AST)

Liver tissues were kept in 10% neutral buffered formalin and embedded in paraffin. Standard methods were used to stain liver samples (5 μm thick) with hematoxylin and eosin (H&E) stain. For collagen staining, the serial sections were deparaffinized and stained with Fast Green FCF (Sigma, St. Louis, MO, USA) for 1 h at room temperature. After that, samples were washed and dehydrated in 100% ethanol and xylene, followed by Permount mounting. Immunohistochemistry (IHC) was performed by using anti-α-smooth muscle actin antibody (Sigma) and anti-proliferating cell nuclear antigen (PCNA) antibody (ABcam, Cambridge, MA, USA). Evaluation of hepatic injury was performed by determinations of serum AST and ALT using a commercial kit from Boehringer Mannheim (Munich, Germany).

### 2.14. Statistical Analysis

Data were analyzed by SigmaPlot version 9.0 (Systat Software Inc., San Jose, CA, USA). Results are presented as mean ± standard deviation (SD) for individual experiments. Statistical differences (experiment vs. control) were calculated by Student’s *t*-test, and *p* < 0.05 was considered as statistically significant.

## 3. Results

### 3.1. Characterization of HA–ADH–PLA

**Figure 2:**
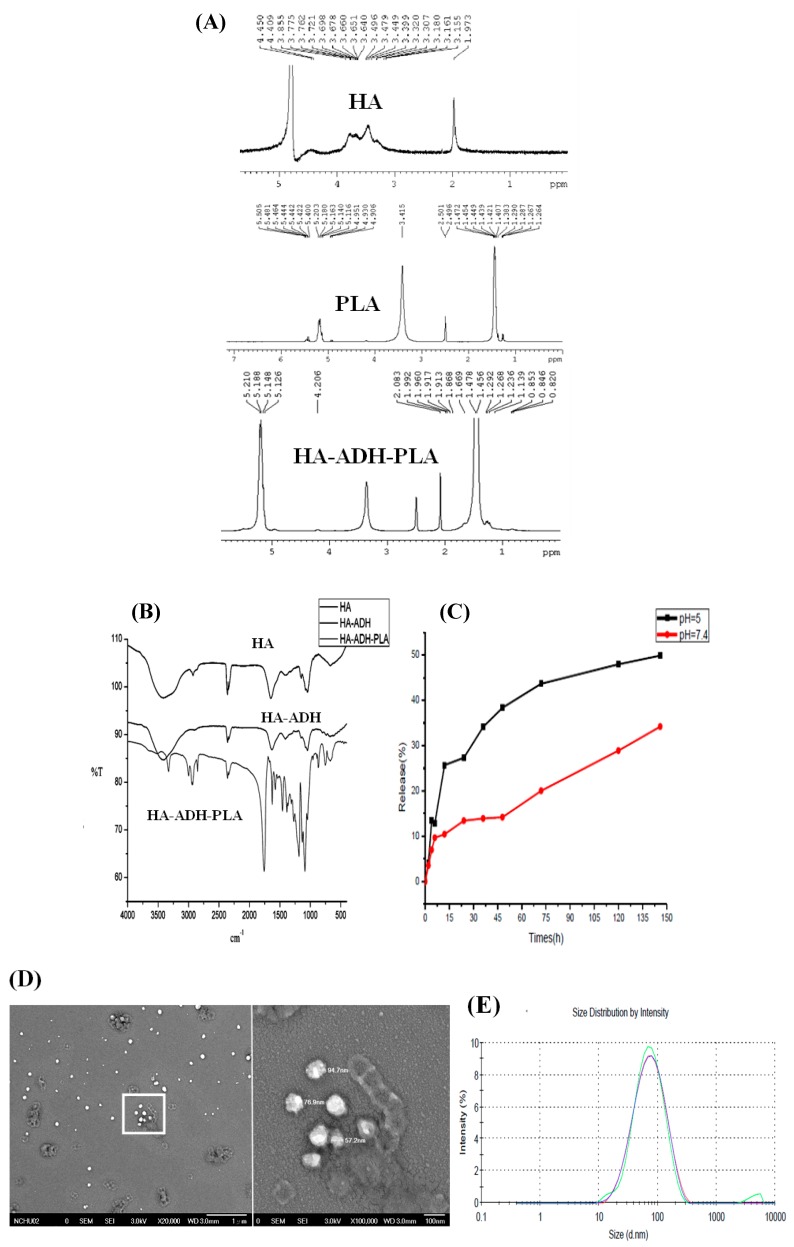
The physicochemical properties of HA–ADH–PLA. The synthesis steps were monitored by (**A**) Nuclear magnetic resonance (NMR) and (**B**) Fourier-transform infrared (FTIR). The drug-releasing profile was measured at (**C**) pH 5.0 and 7.4. Particle sizes were determined by (**D**) scanning electron microscopy (SEM) and (**E**) dynamic light scattering (DLS). The sample was diluted in deionized distilled water (ddH_2_O) and filtered through a 0.22 μm pore size syringe filter for its characterization.

The physicochemical properties of HA–PLA were monitored by proton NMR spectroscopy (^1^H-NMR) and Fourier-transform infrared (FTIR) (Figure 2). The ^1^H-NMR spectrum was δ 1.97 (s, 3H, –NH–CO–CH_3_) for HA; and δ 1.38, δ 1.45 (2d, –O–CO–CH(CH_3_)–O–), and δ 5.1 (m, –O–CO–CH(CH_3_)–) for PLA. HA–ADH–PLA demonstrated a shifted spectrum of δ 2.08 (s, 3H, –NH–CO–CH_3_) for HA; and δ 1.45, δ 1.47 (2d, O–CO–CH(CH_3_)–O–), and δ 5.1 (m, –O–CO–CH(CH_3_)–O–) for PLA, which indicated the successful synthesis of HA–ADH–PLA (Figure 2A). The characteristic FTIR peaks for HA–ADH–PLA (Figure 2B) were 3372 cm^−1^ (N–H stretching of HA), 1078 cm^−1^ (C–N stretching of HA), 1187 cm^−1^ (C–O–C ester of HA), 1614 cm^−1^ (amide of HA), 1300~1500 cm^−1^ (three bands of C–H bending in CH3 groups of PLA); 1757 cm^−1^ (C=O stretching vibrations of PLA), and 2800~2900 cm^−1^ (C–H stretching). These peaks were similar to published results [9,29]. The peaks of HA amide bond shifted from 1614 to 1648 cm^−1^ (HA–ADH amide bond), which indicated the bonding of HA to ADH. Additionally, the appearance of the amide bond peak at 1623 cm^−1^ indicated the formation of anHA–ADH–PLA amide bond. Based on the FTIR and NMR spectra, we confirmed the successful preparation of HA–ADH–PLA.

### 3.2. Characterization of Curcumin-Encapsulated HA–ADH–PLA Nanoparticles (CEHPNPs)

Table 1 lists the physicochemical properties of CEHPNPs. Dissolved HA–ADH–PLA (acetone/ddH_2_O) entrapped curcumin and formed polymeric micelles for drug delivery. The sizes determined by scanning electron microscopy (SEM) and dynamic light scattering (DLS) (Figure 2D,E) for the prepared particles were smaller than 100 nm, and they were average size (60~70 nm). These particles demonstrated zeta potential of approximately 15~30 mV, which suggested a good stability of CEHPNPs. The E.E. was approximately 80%~90%. However, the L.E. seemed unsatisfactory, and ranged from 0.4% to 0.7% (oil/water (O/W): 1/2). To improve L.E, we used polyvinyl alcohol (PVA) as a stabilizing agent to reduce drug leakage from nanoparticles. The addition of different levels of PVA (0.0025%, 0.005%, 0.01%, 0.25%) increased L.E compared to that of the non-PVA treated control group (0.5%) (Table 1). We further changed the O/W (acetone/ddH_2_O) ratio from 1:2 to 1:4. As expected, the L.E. increased approximately 2-fold compared to that of the control group (1.21% vs. 0.5%).

CEHPNPs were designed as an aHSC targeting delivery system, and the spontaneous drug release during circulation should be minimized. The releasing profile showed that there was about a 50% drug release after 150 h incubation at pH 5.0, and 30% at pH 7.4 (Figure 2C). These data suggested that the limited spontaneous release of CEHPNPs at neutral pH assisted these particles to reduce drug loss during circulation, which saved more drug to target cells. In addition, increased release at pH 5, which is the pH of endosomes, enabled CEHPNPs to function efficiently within target cells. These properties make CEHPNPs feasible for the amelioration of hepatic fibrosis.

### 3.3. Evaluation of CD44 Expression on Target Cells

**Figure 3:**
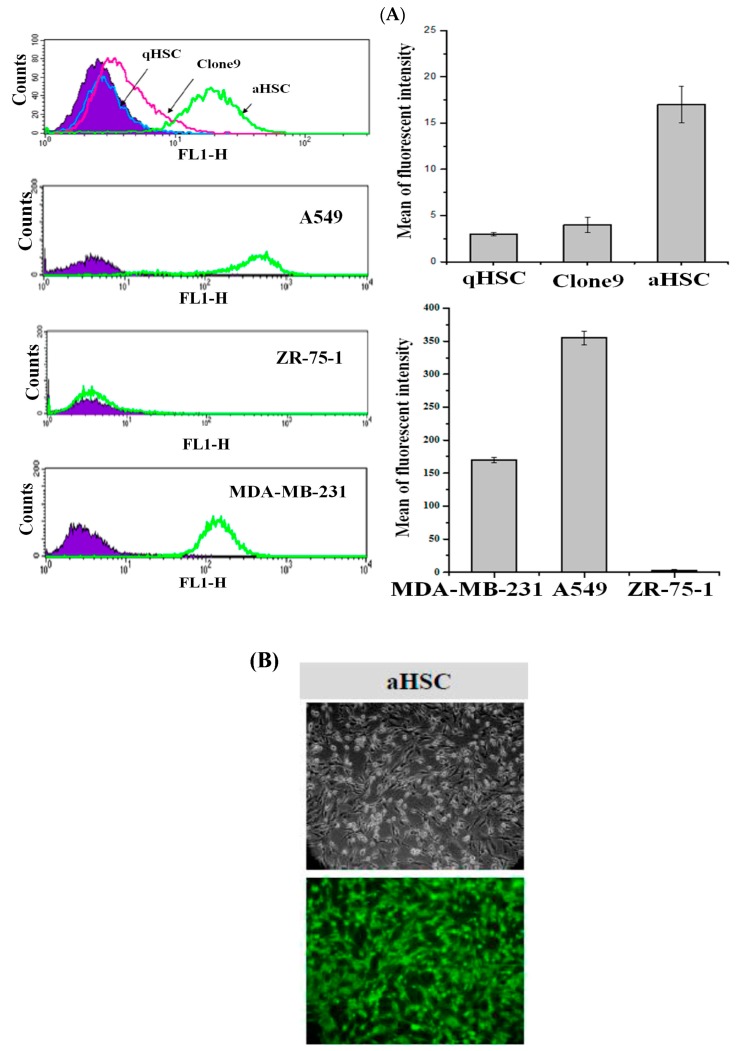
The determination of CD44 expression cells. (**A**) Cells were cultured in Dulbecco’s Modified Eagle’s medium (DMEM) that contained 10% fetal bovine serum (FBS) and 1% antibiotics. It was then grown in a 5% CO_2_ humidified atmosphere at 37 °C for 24 h, followed by labeling with fluorescent anti-CD44 antibody and subjected to flow cytometric analysis; (**B**) activated hepatic stellate cells (aHSCs) were cocultured with coumarin-6-encapsulated HA–PLA nanoparticles for 4 h, followed by fluorescence microscopic analysis. FL1-H: relative intensity of green fluorescent protein florescence.

Elevated CD44 expression elicits diverse cellular activities such as mobility and proliferation [8]. Activated HSCs produce a significant amount of CD44, whereas other liver mesenchymal cells such as qHSCs and epithelial cells do not upregulate the translation of this gene. To evaluate the targeting specificity of the CEHPNP system, the expression of CD44 on rat aHSCs, qHSCs, normal epithelial cells (clone 9 cells), CD44-positive cancer cells (MDA-MB-231 (human breast cancer cells) and A549 cells (human lung cancer cells)), and CD44-negative cells (ZR-75-1 (human breast cancer cells)) were examined by flow cytometry. High levels of CD44 expression were observed in aHSCs, MDA-MB-231, and A549 cells, but low levels of CD44 expression were revealed in CD44-negative ZR-75-1 cells, clone 9, and qHSCs (Figure 3A). Moreover, after 4 h of coincubation of aHSCs with fluorescent HA–PLA nanoparticles (with coumarin-6 encapsulated), a significant number of high-fluorescent cells was observed (Figure 3B). These observations indicated that CEHPNP system could enter liver fibrogenic cells and CD44-positive cancer cells, and had a low tendency to interact with liver normal cells and CD44-negative cells. Additionally, these findings pointed out that the high-efficient targeting ability of HA–PLA nanoparticles on aHSCs, and the potential for amelioration of hepatic fibrosis.

### 3.4. Cytotoxic Effect of CEHPNPs on aHSCs In Vitro

**Figure 4:**
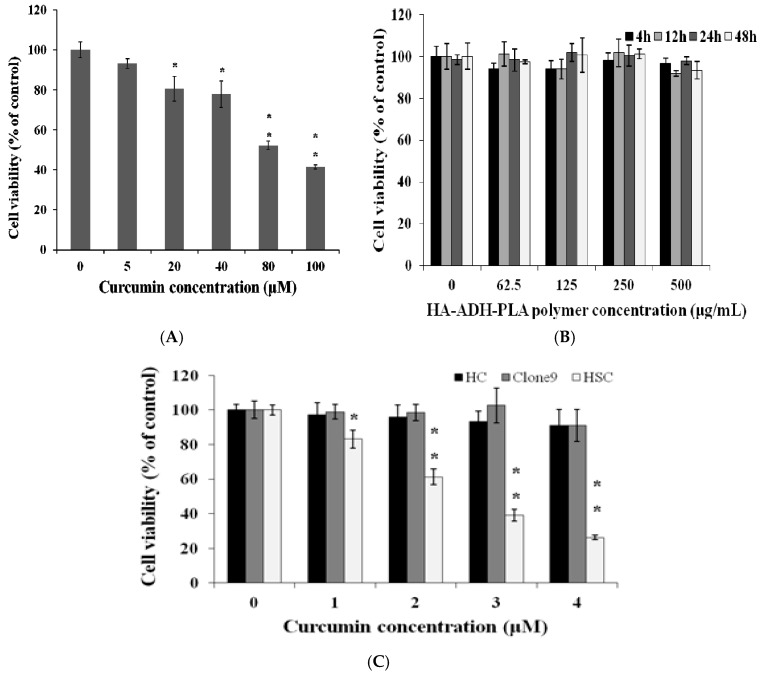
Cytotoxic effects of curcumin-encapsulated HA–PLA nanoparticles (CEHPNPs) on hepatic stellate cells (aHSCs). Activated HSCs were treated with (**A**) free curcumin (0, 5, 20, 40, 80, 100 μM) for 4 h and then replaced with fresh medium and incubated for another 12 h at 37 °C; (**B**) cells were treated with empty HA–PLA carrier (no curcumin) for 4, 12, 24, 48 h; (**C**) CEHPNPs were cocultured with hepatic cells (HC), hepatic epithelial cells (clone 9), and aHSCs for 4 h and then replaced with fresh medium and incubated for another 12 h at 37 °C. The cell viability was determined by MTT (3-(4,5-Dimethylthiazol-2, 5-diphenyltetrazolium bromide) assay. Each bar represents the mean ± SD (*n* = 3). * *p* < 0.05 and ** *p* < 0.01 vs. control group. SD: standard deviation.

We examined the cytotoxicity of free curcumin (5, 20, 40, 80, and 100 μM) (Figure 4A). The cell viability decreased with an increase in curcumin concentration. The IC_50_ (inhibitory concentration at 50%) value of free curcumin on aHSCs was approximately 80.4 ± 2.40 μM. The cytotoxic activity that was obtained agreed with previous reports that curcumin inhibits cell growth and induces apoptosis through modulation of bcl-2/bax protein expression [30]. To evaluate the biosafety of the CEHPNP system, the cytotoxicity of HA–PLA nanoparticles without drug encapsulation was investigated. After 4, 12, 24, and 48 h of coincubation with several concentrations of CEHPNPs (0, 62.5, 125, 250, 500 μg/mL) with aHSCs, we observed no significant cytotoxicity, which indicated that CEHPNPs were safe, bioinert, and held good biocompatibility (Figure 4B). Once encapsulated with curcumin, the CEHPNP system demonstrated significant cytotoxicity toward aHSCs with an IC_50_ value of 2.6 ± 0.17 μM. On the other hand, normal hepatic cells—clone 9, epithelial cells, and primary cultured hepatic parenchymal cells (HC)—showed no significant cytotoxic effect (cell viability above 80%) induced by CEHPNPs, which was probably due to their upregulated catalase activity [31]. Interestingly, CEHPNPs demonstrated a nearly 30-fold strong cytotoxicity (IC_50_: free curcumin vs. CEHPNPs = 80.4 μM:2.6 μM) than that of free curcumin. This huge difference could be due to the solubility of free curcumin, the available concentration in the medium, and the way in which free and encapsulated curcumin enters cells.

### 3.5. Ameliorative Effect of CEHPNPs on Thioacetamide (TAA)-Induced Hepatic Fibrosis

Hepatic fibrosis was induced in male mice by intraperitoneal injection with thioacetamide for 9 weeks (three times per week) [32], and groups were treated with vehicle, free curcumin (150 and 20 mg/kg·wt, i.p.), and CEHPNPs (20 mg/kg·wt, i.p.) for 8 consecutive weeks. Blood analysis (ALT, AST), biopsy (H&E, fast green for collagen analysis, and IHC of α-smooth muscle actin and proliferating cell nuclear antigen for aHSC activity) were conducted for the evaluation of an ameliorative effect.

**Figure 5:**
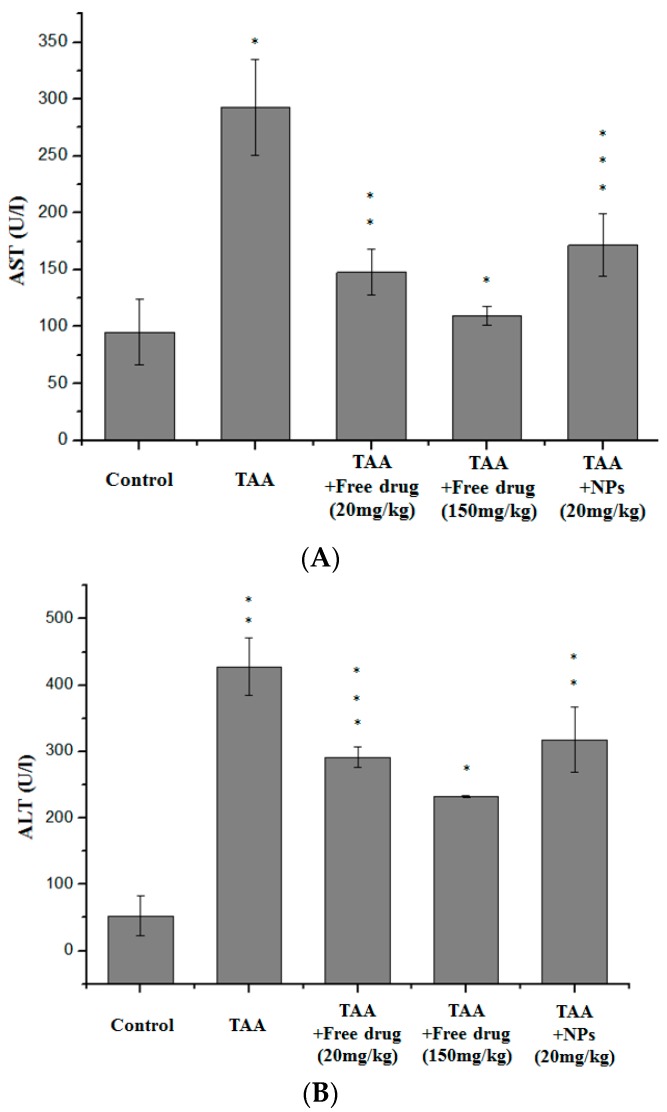
Effect of CEHPNPs on serum (**A**) aspartate aminotransferase (AST) and (**B**) alanine aminotransferase (ALT) and levels in thioacetamide (TAA)-induced liver fibrosis mice. Each bar represents the mean ± SD (*n* = 3). * *p* < 0.05, ** *p* < 0.01 and *** *p* < 0.001 vs. control group. NPs: nanoparticles.

Serum AST and ALT of all curcumin and CEHPEPs treated groups were reduced, although the figures did not decline to the baseline (i.e., control group) (Figure 5). Groups that were treated with free curcumin at 150 mg/kg·wt showed the most significant ameliorative effect. On the other hand, ameliorative results of CEHPNPs-treated group were comparable to the group treated with free curcumin at 20 mg/kg·wt. These findings demonstrated the effectiveness of the CEHPNP system.

**Figure 6:**
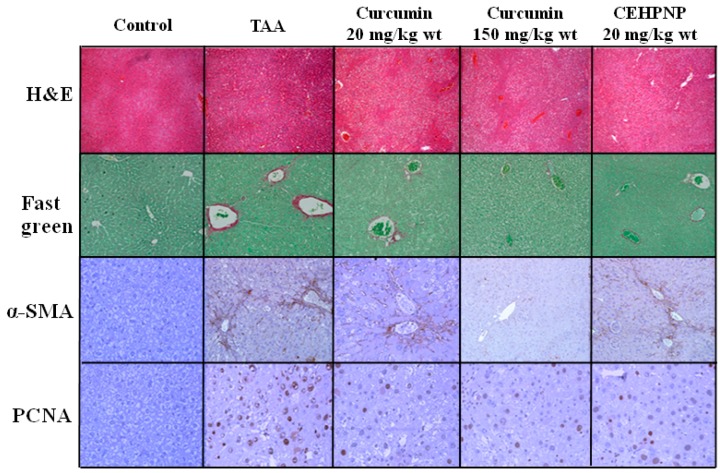
Biopsy results of the CEHPNP system analyzed by hematoxylin and eosin (H&E) staining, fast green staining, and immunohistochemistry (IHC: α-smooth muscle actin (SMA) and proliferating cell nuclear antigen (PCNA)).

For the biopsy (Figure 6), the TAA-treated group showed significant tissue damage as revealed by H&E staining. In the control group, hepatocytes demonstrated visible nucleoli, round nuclei, and chromatin that was dispersed peripherally. No structural changes and cell loss were observed. Group treated with curcumin at 150 mg/kg·wt showed the most significant recovery from TAA-induced fibrosis, reduced production of collagen (fast green staining) andα-smooth muscle actin (IHC), and inhibition of cell proliferation (IHC). Again, the biopsy results of the CEHPNP system and free curcumin (20 mg/kg·wt) were also similar, which further proved the usefulness of the CEHPNP system in fibrosis treatment.

## 4. Discussion

Curcumin has been used widely in preclinical in vitro and in vivo models of hepatocellular carcinoma (HCC) [33]. Although the outcomes of these studies were satisfactory, the curcumin dosage used in these investigations was high due to the disadvantages of low water solubility, poor pharmacokinetics and bioavailability, and physicochemical instability [34]. The dosage of curcumin used in treating xenograft models of HCC was reportedly to be 100 mg/kg/day (i.p.) for 16 days [35] and 200 mg/kg/day [36], or up to 3000 mg/kg/day per os (p.o.) for 14 days [37]. Therefore, efforts have been made to overcome the limitations of the physicochemical properties of curcumin.

Nanoformulation seems an efficient approach for the advancement of curcumin in therapeutic applications. The encapsulation of curcumin by various nanoformulations has been proven successful in enhancing its efficacy in treating cancers, cardiovascular disease, Alzheimer’s disease, inflammatory disorders, and neurological disorders [34]. These nanoformulations include degradable nanoparticles, gold/magnetic nanoparticles, liposomes, polymers, micelles, dendrimers, and nanogels [38], and these could be translated after preclinical and human clinical trials.

HA-based nanoformulations have been used successfully in cancer therapy due to the binding of CD44, an HA receptor that is highly expressed in cancer cells, including cancer stem cells [39]. Activated HSCs also express elevated levels of CD44. Therefore, it is justifiable to adopt HA-based nanoformulation strategies for the amelioration of hepatic fibrosis. Our in vitro results demonstrated that the cytotoxic effect of CEHPNPs outperformed the free curcumin groups. Additionally, CEHPNPs targeted aHSCs specifically, without significant killing of hepatic epithelial and parenchymal cells. Results of animal experiments also revealed that CEHPNPs attenuated TAA-induced hepatic fibrosis effectively, although its therapeutic effect was not as effective as the high-dose group.

Several issues are involved in the effectiveness of CEHPNP in vivo. The surface charge of nanocomposites may significantly affect the interaction of nanoparticles and cell membrane [40]. However, positively charged nanoparticles usually induce a higher level of cellular oxidative stress than that of negatively charged nanoparticles [41], and they disturb the antioxidant system of hepatocytes [42]. Thus, we used negatively charged nanoparticles in the CEHPNP system in this study, which had the advantage of specific targeting of aHSCs and reduced adverse side effects to normal hepatocytes. Moreover, the pharmacokinetic and biodistribution properties of different administration routes, such as intravenous injection (i.v.) or intraperitoneal injection (i.p.), may also significantly influence the success of in vivo experiments. CEHPNPs were designed to target aHSCs, which are located in the perisinusoidal space between sinusoids and hepatocytes. It would be preferable to use intravenous injection for CEHPNPs instead of using intraperitoneal injection.

The administration of nanoparticles by intraperitoneal injection may result in low bioavailability and accumulation in peritoneal membranes [43]. Peritoneal membranes that comprise mucus-secreting cells serve as barriers to shield the abdominal cavity and to block substances to enter the blood stream. These mucus-producing tissues act as a barrier to prevent nanoparticles from entering into blood circulation. As a result, based on our findings from animal experiments, the therapeutic effect of nanoparticles on attenuation of liver fibrosis was abated. The penetration of the mucus lining by low-molecular-weight polyethylene glycol molecules has been suggested [44]. It would be advantageous to use this strategy to improve our CEHPNP system.

Finally, the encapsulation efficiency (E.E.) and loading efficiency (L.E.) of the CEHPNP system could also be the reason that led to the limited results in animal experiments. For high encapsulation efficiency, factors such as low solubility of polymer in organic solvent, high solubility of organic solvent in water, high concentration of polymer, low ratio of dispersed phase (DP) to continuous phase (CP), and rapid rate of solvent removal have been proposed [45]. The main idea of E.E. is to precipitate polymer on the surface of DP rapidly, which prevents drug loss (diffusion) into CP. Usually, the E.E. was above 90%. Our results indicated that there were only two sets of E.E. above 90% (control group: 93.7%, group D: 94.7%). The addition of PVA can stabilize the primary emulsion. As seen in group D, the E.E. increased to 94.7% with the addition of PVA at 0.25%. However, the L.E. values of these two groups were quite low. We then modified the ratio of DP to CP from 1:2 to 1:4 (acetone to water). As seen in group E, the L.E. increased to 1.2%, but E.E. was decreased. These results suggested that the preparation conditions should be optimized further to obtain high E.E. and L.E. values. The drug uptake levels of liposome can achieve up to 0.29:1 (drug:lipid, wt/wt) [46].

## 5. Conclusions

In the present study, we developed HA–PLA nanoparticles that were encapsulated with curcumin, a natural hepatic protective agent in turmeric, to elicit cytotoxicity toward aHSCs and attenuate thioacetamide (TAA)-induced rat hepatic fibrosis. Our results indicated that the particle size of curcumin-encapsulated HA–PLA nanoparticles (CEHPNPs) was approximately 60~70 nm. This drug system induced significant aHSC cell death without affecting quiescent HSCs, hepatic epithelial cells, or parenchymal cells. This system reduced drug dosage without sacrificing therapeutic efficacy. The cytotoxicity IC_50_ value of this system was approximately 1/30 to that of the free drug-treated group in vitro. Additionally, the therapeutic effects of CEHPNPs were as effective as the group treated with the same curcumin dose intensity in vivo. The advantages of superior biosafety and a satisfactory therapeutic effect suggest that CEHPNPs hold great potential to be applied in treating hepatic fibrosis.

## Figures and Tables

**Table 1:** Preparation of curcumin-encapsulated hyaluronic acid-polylactide nanoparticles (CEHPNPs).

Group	Acetone/ddH_2_O (mL)	PVA% (*W*/*V*)	Particles Size (nm) ^a^	E.E. ± SD (%) ^a^	L.E. ± SD (%) ^a^	Zeta Potential (mV)
Control	1/2	n/a	69.2 ± 0.48	93.7 ± 0.49	0.5 ± 0.05	−37.8
A	1/2	0.0025	58.5 ± 0.04	79.5 ± 2.12	0.6 ± 0.12	−29.6
B	1/2	0.005	59.3 ± 1.26	89.1 ± 0.32	0.4 ± 0.04	−27.6
C	1/2	0.01	57.7 ± 0.55	89.1 ± 0.74	0.7 ± 0.03	−15.9
D	1/2	0.25	55.7 ± 0.39	94.7 ± 0.06	0.4 ± 0.02	−15.6
E	1/4	n/a	54.7 ± 0.64	82.6 ± 2.18	1.2 ± 0.08	−29.6

**^a^** The drug/polymer ratio (mg/mg) of all groups was 0.2/15. ddH_2_O: deionized distilled water; E.E.: encapsulation efficiency; L.E.: loading efficiency; n/a: not applicable; PVA: polyvinyl alcohol; SD: standard deviation.
